# A reassessment of the infra-species diversity patterns in the wine-associated *Oenococcus oeni*

**DOI:** 10.3389/fmicb.2025.1657712

**Published:** 2025-09-24

**Authors:** Florencia Oviedo-Hernandez, Magali Bou-Deleris, Sibylle Krieger-Weber, Florian Claisse, Jovnna Dereme, Claire Le Marrec, Olivier Claisse

**Affiliations:** ^1^Université Bordeaux, UMR1366 Oenologie, Bordeaux INP, Bordeaux Sciences Agro, ISVV, Villenave d’Ornon, France; ^2^Lallemand SAS, Blagnac, France

**Keywords:** malolactic fermentation, infra-species diversity, accessory genome, mobile elements, transposon

## Abstract

*Oenococcus oeni* is the predominant species of lactic acid bacteria in wine, where it carries out malolactic fermentation (MLF), which helps to ensure and preserve the quality of the wine. Today, existing combinations of grape varieties, soil composition, fluctuating climatic parameters, and specific technical processes implemented by wineries lead to incredibly varied wine compositions that pose challenges for spontaneous MLF. Commercial starter cultures have been developed for use as inoculants. However, their effectiveness in ensuring consistent and reliable MLF is also limited in modern wines. The selection process must therefore adapt to these new challenges, which means expanding current portfolios by selecting more robust bacteria from wines that are more varied in terms of grape varieties and chemical constraints. We have assembled a set of 21 wines produced in Europe from different grape varieties, with varying and, in some cases, extreme ethanol contents, total polyphenolic indices, and pH levels. The isolation and MLVA typing of 385 dominant colonies were combined with whole-genome sequencing of 48 representative strains, and we observed several strains with unique accessory genomic content. Different selective pressures led to the formation of groups of genetically related individuals, particularly in white and rosé wines with moderate ethanol content. However, cohabiting strains with contrasting genetic profiles were also observed in some red wines. Our data highlight the complexity of the factors involved in population heterogeneity and raise the possibility that this phenomenon may increase fitness through diversification of strategies or division of labor in specific production environments.

## 1 Introduction

The genus *Oenococcus* was proposed by Dicks et al. (1995) to group together the lactic acid bacteria (LAB) of the species *Leuconostoc oenos*. These bacteria play a key role in the production and quality of grape wine through the bioconversion of malic acid into lactic acid and CO_2_, a process also known as malolactic fermentation (MLF). The *O. oeni* species is associated with other habitats such as apple ciders and kombuchas. More recently, three sister species (*O. sicerae*, *O. kitaharae*, and *O. alcoholitolerans*) have been introduced into the genus. Their members are adapted to harsh environments found in overripe fruits and sugar-based fermentation processes used to produce beverages (ciders, liqueurs, kombuchas, water kefirs) and bioethanol (Badotti et al., 2004; Endo and Okada, 2006; Cousin et al., 2019). Oenococci may also be present in coffee pulp, honeycomb, and brine-type fermented foods (Lorentzen and Lucas, 2019). Overlapping niches between species have been detected, with water kefir promoting the growth of the four species described (Barchi et al., 2023).

Twenty years after the sequencing of the first complete genome by Mills et al. (2005), the approach has developed considerably within the *Oenococcus* genus. Due to its impact on the wine industry, much research effort has been devoted to the species *O. oeni*, and researchers have established the phylogenetic profile of hundreds of strains collected from wines and their production environments. This work has provided essential information on gene function and genome evolution. The data also highlighted signatures in bacterial genomes and made it possible to trace the specific evolutionary trajectory of *O. oeni* strains during their domestication for wine production, compared to cider and kombucha (Rudolf et al., 2024). Four phylogroups are currently described in the species, and phylogroup A exclusively includes strains that are predominant in wine (Campbell-Sills et al., 2015; Campbell-Sills et al., 2017).

Another challenge is to understand the dynamics within the indigenous populations of *O. oeni* at the cellar level, highlighting regional variations and their impact on wine quality (Lorentzen and Lucas, 2019). From a chemical standpoint, the transition from grape berry to wine is a particularly complex process. Major environmental changes lead to non-linear dynamics, causing substantial variations in the cultivable population and genetic diversity throughout fermentation. There are two growth periods for *O. oeni* during winemaking, first when berries are crushed and then during MLF. The two periods are separated by a latency period during alcoholic fermentation (AF). In this multi-step process, species must adapt to various types of stress, including high osmolarity, low pH, nutrient depletion, the presence of polyphenolic compounds (PC), sulfite doses, and increasing ethanol content. Another level of complexity is due to the variable kinetics of stress imposition throughout production, its duration, and its intensity. Acidity is one of the first stresses for bacteria and results from the release of organic acids by grapes during crushing, with L-tartaric, L-malic and citric acids contribute significantly to the reduction in pH. Malic acid is also responsible for a concomitant increase in osmolarity, along with sugars and potassium (Winkler and Knoche, 2018). The medium gradually becomes enriched with PC, which are transferred from the grape skins into the must during the pre-fermentation maceration of red wines. As the alcohol content increases during AF, these compounds also become more soluble (Watrelot and Delchier, 2025). Therefore, the AF stage may represent a selective bottleneck for the *O. oeni* population and select strains from phylogroup A (Balmaseda et al., 2023). AF is followed by MLF, which usually starts when LAB reach a viable cell population of approximately of 10^6^ CFU/mL. A successful MLF reflects the non-limiting presence of nutrients and the ability of the indigenous population to survive and adapt to the cumulative effects of all the stress factors to which it has been subjected during the previous stages. It is important to note that the chemical variables in the composition of the must/wine are also linked to factors related to winemaking practices, which are not uniform among winegrowers (González-Arenzana et al., 2023). The entanglement of factors is likely to make each wine composition and bacterial selection process unique. In line with this idea, observational studies in wineries often show variations in MLF duration. The step can last anywhere from a couple of weeks to 3 or 4 months, with frequent cases of sluggish or stuck fermentation being reported worldwide (Bech-Terkilsen et al., 2020).

Understanding selection scenarios and the link between genetic variations in favored bacterial genotypes and wine variables and/or cellar practices during spontaneous fermentation has been the subject of extensive research. Newfound knowledge is expected to guide the design of effective MLF starters, adapted to modern wines, which are more difficult to ferment. Remarkably, and unlike other Lactobacillaceae, a constitutive hypermutable status has been reported for the entire *Oenococcus* genus, due to the loss of the *mutS*-*mutL* gene responsible for repairing DNA mismatches (Marcobal et al., 2008). In their respective ecosystems, genotypes with increased mutation rates, known as hypermutators, have many advantages, enabling them to survive in complex and stressful nutritional conditions. The selection of the most fit alleles at specific loci is likely to be driven by the selection of adaptive and innovative mutations, recombination, and/or the acquisition of functional alleles through horizontal transfer (Margalef-Català et al., 2017; Betteridge et al., 2018; Van den Bergh et al., 2018; Contreras et al., 2023; Julliat et al., 2023; Zheng et al., 2024). The genetic basis of stress adaptation is important in *O. oeni*. Exposure of the bacterium to multiple stressors triggers a range of responses, with a large repertoire of annotated genes involved in adaptation to wine-related stresses. These are related to essential bacterial metabolisms, the synthesis of stress response proteins, and the maintenance of cellular integrity, and include several genes acquired by horizontal transfer (Bon et al., 2009; Rudolf et al., 2024).

New paradigms are emerging in the microbial ecosystems of natural foods, suggesting that fermentation performance is linked not only to the individual response of members, but also to their social behaviors, leading to the emergence of functional consortia (Louw et al., 2023). It is important to note that ecological interactions between members vary depending on environmental stress gradients (Wall, 2016; Choudhary et al., 2023). Wine is no exception, and interactions between yeasts and *O. oeni* are likely to have an impact on the latency phase between AF and MLF (Englezos et al., 2022; Luo et al., 2024; Balmaseda et al., 2024). This area of research is also increasingly incorporating infraspecific trait variability and its possible role in community assembly and fitness (Capozzi et al., 2021). To illustrate this, a few studies have recently shown that MLF is dominated by up to 10 *O. oeni* genotypes in red wines (Garofalo et al., 2015; El Khoury et al., 2017; Chaïb et al., 2022a). In natural environments, individuals with diverse conspecific genotypes may form kin groups and contribute to metabolic cooperation and fitness during fermentation. The existence of cooperative groups also implies the existence of cheaters, as well as the ability of cooperators to compete with and eliminate them from the group, with the transfer of mobile genetic elements (MGEs) being used as a mechanism to reinforce cooperation (Niccum et al., 2020; Lee et al., 2022; Lin et al., 2023; Weltzer and Wall, 2023).

In this study, 385 low-passage isolates *O. oeni* isolates were collected from 21 wines of all types (red, rosé, and white) produced in European countries during the 2021 vintage. We included several grape varieties, of which Pilzwiderstandsfähig (PIWI), which are highly resistant to fungal diseases, and Malbec, a historic grape variety from southwestern France and other wine-growing regions around the world (Griset and Laborie, 2016). Currently, the strains associated with these grape varieties remain underrepresented in general databases, including the collection of the Institute of Vine and Wine Sciences (ISVV) in Bordeaux. Our objectives were to elucidate intraspecific genomic diversity in order to understand how natural selection shapes the genome of *O. oeni*. Our study focused particularly on the species’ accessory genome and MGE repertoire, as well as on predicting the potential functions of certain mobile genes in order to provide inferences about the ecological importance of these functions in strain lineages and wine composition, which could contribute to understanding the dynamics of MLF. In addition, we examined the richness of populations and the genetic proximity of resident strains in different wines (Supplementary Figure S1).

## 2 Materials and methods

### 2.1 Wine collection and analyses

#### 2.1.1 Origin of samples

A total of 21 red, white, and rosé wines were sampled from various wineries located in Europe (France, Spain, the Netherlands, Belgium, and Germany) during the 2021 vintage (Supplementary Table S1). Winemakers did not use commercial bacterial starter in order to promote MLF and checked that AF was completed. The wines were then sampled from the fermentation tanks (250 mL) before the addition of SO₂. Samples were transported by express road transport. They were named using a code where the letter “W” stands for “wine,” followed by a randomly assigned number; for example, “W2” refers to wine number 2 (Supplementary Table S1).

#### 2.1.2 Measurement of wine chemical parameters

The standard oenological parameters (ethanol, pH, malic and lactic acid) were provided by each producer. The latter values were confirmed upon receipt by additional sampling of 15 mL of each wine at room temperature, whose parameters were measured using the FOSS OenoFoss™ FTIR multiparametric analyzer, applicable to wines and musts (Foss France SAS, Nanterre, France). The same device was used to measure the total polyphenolic index (TPI). Wines that did not contain lactic acid upon receipt were incubated at 25 °C in their original 250 mL bottles and resampled periodically until L-lactic acid production was detected by FTIR OenoFoss™.

Agglomerative hierarchical clustering analysis (HCA) was performed on the enological parameters (ethanol (V/V), pH and TPI) to identify similarities or dissimilarities between the considered 21 wine samples. XLSTAT (Addinsoft, Version 2024.1) was used to generate Principal Component Analysis (PCA). Statistics were performed in RStudio (version 1.4.1717, RStudio Team, Boston, MA, United States) using ggpubr (R package version 0.4.0).

#### 2.1.3 Bacterial counts

All wines were then subjected to bacterial cell counting. Ten-fold serial dilutions were prepared in saline buffer (NaCl 0.9%) and spread on Red Grape Juice (RGJ) agar plates in duplicate. The medium contains 25% (V/V) of commercial red grape juice (Reflets de France; ~ 2 g glucose and 2 g of fructose), 0.5% (W/V) of yeast extract, 0.1% (V/V) of Tween 80 and 2.5% (W/V) of bacteriological agar and was adjusted to pH 4.8 (Chaïb et al., 2019). Media were supplemented with 0.1 mg/mL Delvocid^®^ to inhibit the growth of yeasts and molds. Plates were incubated in jars under anaerobic conditions using GasPak™ sachets (Anaerocult, Merck, Grosseron, France) for 7–10 days at 25 °C until the appearance of colonies forming units (CFU). Dilutions showing 30–300 CFUs were retained for counting.

#### 2.1.4 Assessment of MLF progress

The absence of lactic acid, measured by FTIR, indicated that MLF had not begun. Conversely, the presence of lactic acid and malic acid indicated that MLF was underway. When the malic acid concentration was below 0.5 g/L, MLF was considered complete. These parameters are usually associated with a bacterial concentration greater than 10^6^ CFU/mL.

### 2.2 Isolation of dominant colonies and species identification

Upon enumeration, the countable plates were retained for each wine and colonies (15–19) were randomly selected (Chaïb et al., 2022a). They were purified by two successive passages on RGJ agar in order to limit spontaneous loss of mobile genetic elements (Favier et al., 2012).

Fresh colonies were picked and species identification was performed by MALDI-TOF MS (matrix assisted laser desorption ionization-time of flight mass spectrometry) as described by Windholtz et al., 2021. Spectra were collected using a Microflex LT/SH (Bruker Daltonics GmbH & Co, Bremen, Germany) mass spectrometer equipped with a nitrogen laser (lambda = 337 nm) at a laser frequency of 60 Hz operating in linear positive ion detection mode under MALDI Biotyper Compass 3.0 and FlexControl 3.4 (Bruker Daltonics GmbH & Co, Bremen, Germany). Mass spectra were acquired in the range of 2,000–21,000 Da for each sample analyzed for species level microbial identification.

### 2.3 VNTR genotyping of *Oenococcus oeni* isolates and detection of prophages

All identified *O. oeni* isolates were subsequently grown in MRS broth pH 4.8 (Difco, Fischer Bioblock Scientific, Illkirch, France). To extract and store genomic DNA, cultures were spotted onto Whatman FTA Cards (QIAcard FTA CloneSaver®) following manufacturer instructions (Qiagen, Germany), and the rest was added with 30% (V/V) glycerol and stored at −20 °C. DNA quality on FTA cards proved reliable for PCR. A 1.2 mm FTA disk was punched out of the FTA card using a Uni-Core Punch (Qiagen, Germany). It was cleaned twice with TE Buffer (Tris HCl 10 mM; EDTA 1 mM pH 8) and introduced in the PCR reaction mixture (Chaïb et al., 2022a).

The standard 5-locus variable number of tandem repeat (VNTR) analysis (MLVA) included PCR amplification followed by the sequencing of the amplicons by MWG Eurofins Operon (Ebersberg, Germany) as earlier described (Claisse and Lonvaud-Funel, 2014). A Biorad i-Cycler was used for the amplification reactions, which were achieved in a 25 μL volume using the Bio-Rad Taq PCR Master Mix kit and 0.2 mmol/L of each primer. The processing of VNTR typing data and comparisons with profiles from the ISVV collection were performed using Bionumerics (Version 8, Applied Maths, Inc., Saint-Martens-Latem, Belgium). Isolates with distinct profiles were considered as distinct strains.

A multiplex PCR assay was developed for the simultaneous detection and differentiation of the four integrase genes (*int*) harbored on prophages. Fragments of 163 bp (*intA*), 254 bp (*intB*), 438 bp (*intC*) and 343 bp (*intD*) were obtained. The primer sequences of the target genes (*intA* to *intD* and *mle*) are given in Supplementary Table S2. PCR consisted in an initial denaturation at 95 °C for 5 min, and 40 cycles, each consisting of a denaturation step (30 s, 95 °C), an annealing step (30 s, 52 °C), and an extension step (20 s, 68 °C). PCR products were compared by using a MultiNA system (Microchip Electrophoresis System for DNA/RNA Analysis, Shimadzu).

### 2.4 Assessment of intraspecies diversity in wine samples

Two parameters were used to compare the diversity of strains in each wine. The strain richness (S) represents the total number of strains (isolates showing distinct VNTR profiles) identified in a wine sample. The Shannon’s index (H) expresses the diversity of an environment as a function of the number individuals (strains) and their abundance in a sample. It was calculated using the formula *H* = $-\overset{n}{\underset{i=1}{\sum }}p_{i}\ln pi$, where *i* is a strain of the sample and *pi* is the proportion of a given strain (number of individuals of *i*/number of total individuals). The maximum values observed in complex ecosystems are around 4.5 (Frontier et al., 2008).

### 2.5 Whole genome sequencing and bioinformatic analyses

A volume of 2 mL of a late exponential phase culture in MRS broth pH 4.8 was centrifuged and the cell pellet washed twice with sterile water. DNA extraction was carried out using the Wizard® Genomic DNA Purification Kit (Promega, France) with some modifications. Briefly, cells were resuspended in 600 μL of EDTA 50 mM added with lysozyme (10 mg/mL) and incubated at 37 °C for at least 1 h. Samples were centrifuged (5,000 × g for 2 min) and the pellet resuspended in 600 μL of Nuclei Lysis Solution. The mixture was incubated at 80 °C for 5 min, and cooled down to room temperature. A volume of 3 μL of RNase solution was added and samples were incubated at 37 °C for 1 h. The RNase-treated samples were added with 200 μL of protein precipitation solution and incubated on ice for 5 min. Samples were centrifuged at 18,000 × g for 15 min. The supernatants were transferred to tubes containing 600 μL of isopropanol. DNA was collected by centrifugation (18,000 × g for 15 min) and washed twice with a 70% (V/V) ethanol solution, and resuspended overnight in nuclease-free water.

Whole-genome sequencing was performed at the Genome-Transcriptome facility of Bordeaux. DNA libraries were prepared using the Nextera XT DNA library preparation kit (Illumina, San Diego, CA). Genomic DNA was sequenced using an Illumina MiSeq using 2 × 250 bp paired end libraries. Bacterial isolate genomes from paired-end reads were assembled with Shovill 1.1.0 (https://github.com/tseemann/shovill) with the Skesa 2.4.0 assembler. All assemblies are annotated using Prokka v1.14.6 (Seemann, 2014). The genome sequences were submitted to GenBank and their accession numbers are given in Supplementary Table S3.

The *in silico*-translated protein sequences were used as queries to search for sequence homologs in the non-redundant protein database at the National Center for Biotechnology (including the viral genome database). Deduced proteins were searched for function using BLAST v2.10.0 and a cutoff E-value of 0.001. Searches for distant homologs were performed using HHpred (Söding et al., 2005) against different protein databases, including PFAM (Database of Protein Families), PDB (Protein Data Bank), CDD (Conserved Domains Database), COG (Clusters of Orthologous Groups) and PHROGs (Prokaryotic Virus Remote Homologous Groups) which are accessible via the HHpred website.

Comparisons of phage genomes were conducted using the Genome-BLAST Distance Phylogeny (GBDP) method using VICTOR (http://ggdc.dsmz.de/phylogeny-service.php) (Meier-Kolthoff and Göker, 2017). The resulting intergenomic distances were used to infer a balanced minimum evolution tree with branch support via FASTME including SPR post-processing for each of the formulas D0, D4 and D6, respectively. Branch support was inferred from 100 pseudo-bootstrap replicates each. Trees were rooted at the midpoint and visualized with FigTree as already described (Barchi et al., 2022).

For bacterial genomes, the Pyani application (https://github.com/widdowquinn/pyani) (Pritchard et al., 2016) was used to calculate the average nucleotide identity (ANI) distance between individuals in terms of global nucleotide similarity. For phylogenomic analyses, data formats were adapted. Programs for calculating ANI genomic distances usually require an output corresponding to a similarity matrix in the form of a table. The latter are not compatible with phylogeny analysis software such as MEGA (https://www.megasoftware.net/) (Tamura et al., 2021). In order to connect the pipelines, an in-home script was developed to read the ANI similarity matrix and transform the data into distances to accommodate to the required MEGA format.

PPanGGOLiN v1.2.74 was used with default parameters for pangenome analysis of the *O. oeni* genome assembly panel (Gautreau et al., 2020). PPanGGOLiN generates a core gene set based on a Partitioned Pangenome Graph (PPG), which integrates information about protein-coding genes and their genomic neighborhood. Additional tools used to characterize the mobilome were IS Finder (Varani et al., 2011) and geNomad (Camargo et al., 2023).

## 3 Results and discussion

### 3.1 Assembly of a set of wines with distinct physico-chemical parameters

The study was conducted on a group of 21 wines, divided into three types as follows: reds (*n* = 11), whites (*n* = 6), and rosés (*n* = 4). All were produced during the 2021 vintage in five European countries, from several grape varieties, including Malbec and fungus-resistant varieties (PIWI). Three parameters traditionally monitored during winemaking (pH, ethanol, and total polyphenolic index or TPI) were measured by the partner wineries and confirmed upon receipt of the samples at the laboratory (Supplementary Table S1).

**Figure 1:**
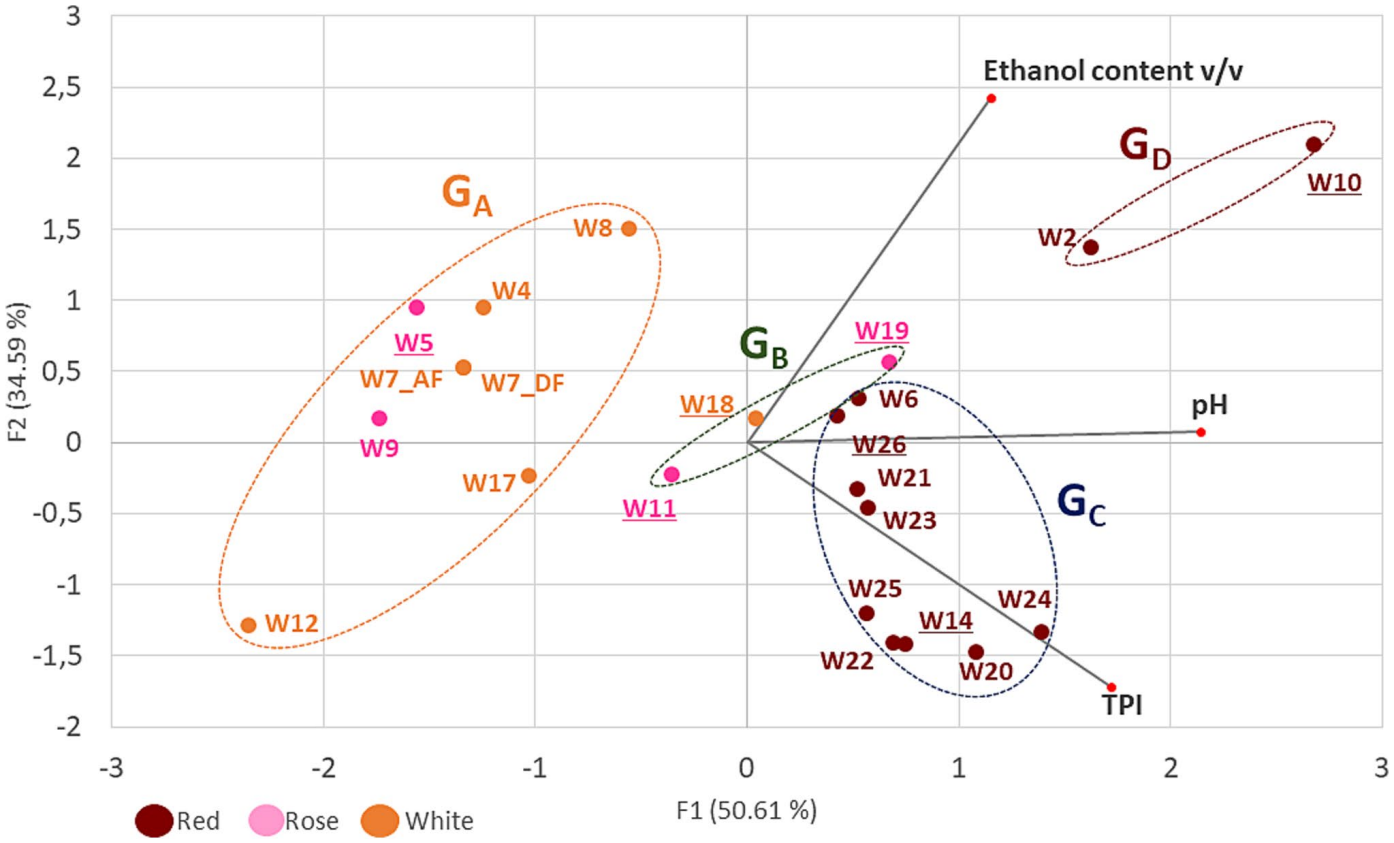
Principal component analysis (PCA) of three enological parameters (ethanol content, pH and TPI) of 21 wines. Red, white and rosé wines are represented in brown, orange and pink characters, respectively. Wine groups (G_A_ to G_D_) are represented by ellipses, which were determined by an agglomerative hierarchical clustering analysis (HCA). Major axes of variation, F1 and F2, explain 50 and 34% of chemistry variation, respectively. The wines with underlined numbers showed slowed stuck or uncomplete MLF.

Principal component analysis was used to identify clustering patterns among the wine samples and four groups were thus identified and named G_A_ to G_D_ (Figure 1). Groups G_A_ and G_B_ contained only white and rosé wines. Group G_A_ included the most acidic wines (pH < 3.6). The ethanol content ranged from 9 to 14% and the TPI values were low (<10). The three wines in group G_B_ had slightly higher TPI values (10 to 19), an average ethanol content (11 to 12%) and a rather high pH (> 3.75). Also from Figure 1, red wines with higher TPI (34–84) clustered apart in groups G_C_ and G_D_. Samples with moderate acidity (pH 3.8 to 3.5) and moderate ethanol content (11.4–12.2%) were grouped into the G_C_ group. The G_D_ group included samples W2 and W10, which had fairly high pH values and high ethanol content (>14.3%). It should be noted that the highest ethanol content was in W10 (16.6%), and corresponds to the average content of approximately 14–16% previously reported for wines made from the Monastrell grape variety (Griset and Laborie, 2016). These parameters represent challenges for modern oenology and are currently prompting researchers to seek solutions to reduce the potential risks of deviation. Finally, wines W5, W24, and W10 are interesting in the set of samples tested because they have an extreme value for one of the three constraints monitored in the study (pH 3.1, IPT 82, and 16.6% ethanol) and are distributed, respectively, in groups G_A_, G_C_, and G_D_.

All wines were announced as being in or at the end of MLF. This was confirmed in most samples with the presence and load of LAB, as well as malic and lactic acid contents. As shown in Supplementary Table S1, concentrations of 10^6^ CFU/mL and above were observed in 17 of 21 wines. This population level was conducive to effective malic degradation in 13 wines which were distributed in G_A,_ G_C_ and G_B_ groups (Figure 1). MLF was triggered, but not completed in the four additional samples (W10, W11, W14, and W26), as evidenced by the detection of both lactic acid and residual malic acid. Unlike these 17 wines, the remaining samples (W5, W7, W18, and W19) had low bacterial counts (10^1^–10^3^ CFU/mL). This was not expected for sample W5 which had fully completed the conversion of malic acid into lactic acid. The data was interpreted as a sign of early MLF in W5, which likely occurred well before the sample was received in the laboratory. Prolonged incubation in a depleted environment in the cellar may have further impaired cell viability. However, samples W7, W18, and W19 still had high malic acid content and very low lactic acid concentration. These three wines were incubated at 25 °C in the laboratory. Bacterial growth was observed in sample W7, causing the pH to increase from 3.25 (sample W7_AF) to 3.42 (sample W7_DF). Conversely, incubation of samples W18 and W19 had no effect on the start of MLF.

In conclusion, differences in wine composition and degree of MLF completion were observed among the samples tested. In particular, none of the wines in the G_B_ group underwent complete MLF. Samples W18 and W19 showed stuck MLF, while the process was incomplete in the case of W11, suggesting that specific combinations of stress factors inhibited bacterial growth in these samples collected in Belgium and the Netherlands. In addition to pH, ethanol and PC levels, these samples probably contained other specific growth-limiting factors, such as fluctuating nutrient supplies, the presence of inhibitors, and/or were the result of specific winemaking practices, such as low temperatures during fermentation.

### 3.2 *Oenococcus oeni* is the dominant species driving MLF in the tested wines

RGJ agar plates which were selected for accurate counting were kept and used to collect 15–19 colonies per wine. A total of 385 colonies were selected from all wines and subjected to MALDI-TOF MS analysis. All isolates were identified as *O. oeni*, indicating that this species was the dominant microbial group at the time of sampling in all wines. No other species were found in the wines sampled using RGJ medium, which is a permissive medium for most LAB species associated with wine. This demonstrates once again that *O. oeni* is the species best adapted to wine conditions.

**Figure 2:**
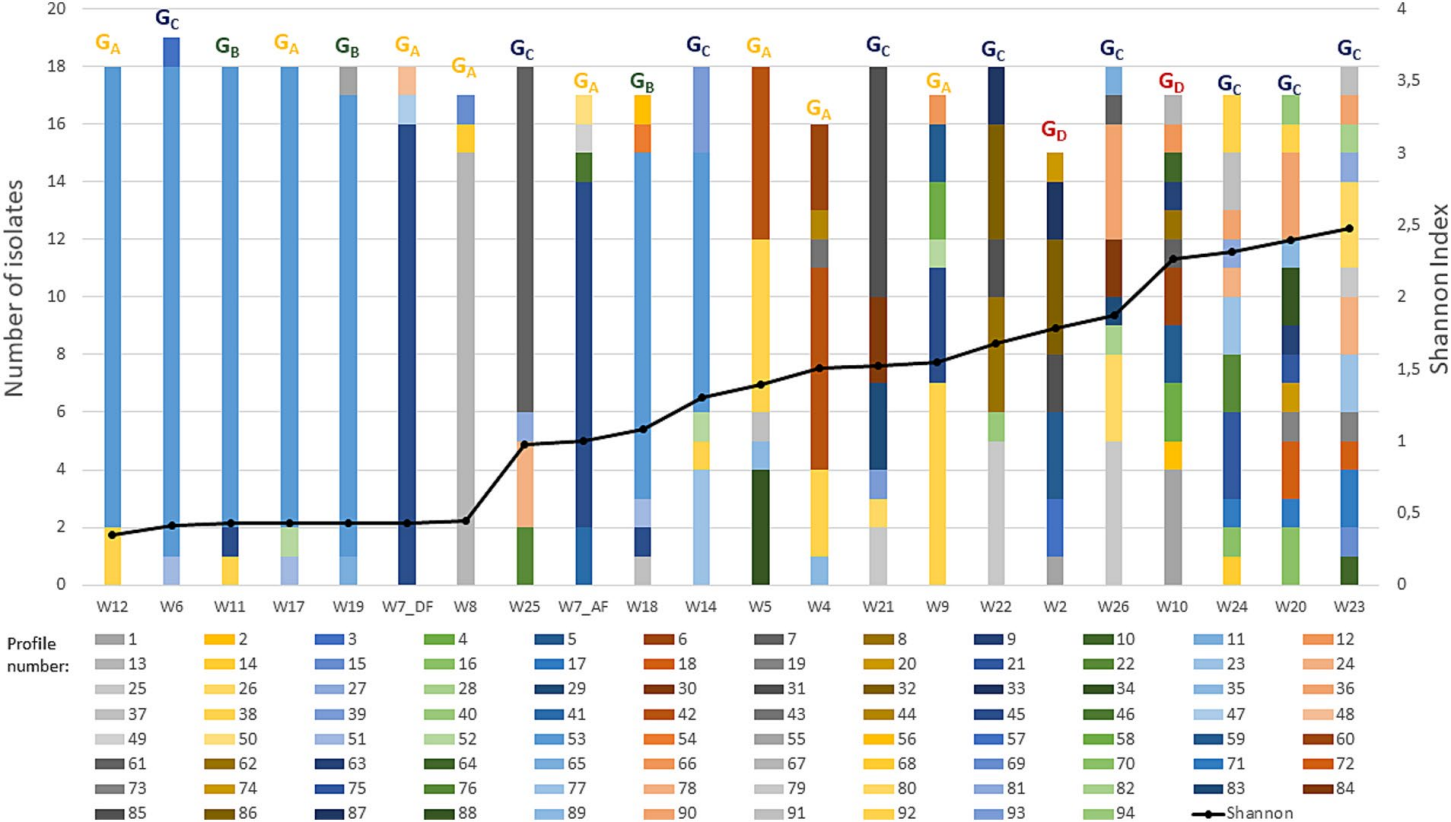
Genetic diversity of *O. oeni* isolates at the wine’s scale. A number of 15 to 19 isolates were isolated in 22 samples from 21 wines. The samples were analyzed using VNTR, generating 94 VNTR profiles. Histograms illustrate the distribution of these profiles per wine. Each profile is represented by a different color within the histograms. The black curve represents the alpha diversity, as measured by the Shannon-Weiner diversity index at sub-species-level resolution. The strains membership to each wine groups (G_A_ to G_D_) previously identified based on the principal component analysis on enological parameters are also represented with the same color code.

A multilocus fragment typing approach comprising five variable number tandem repeat (VNTR) loci was successfully used to study the genetic diversity of the isolates. The analysis characterized 94 distinct profiles. Their distribution, frequency, and redundancies among wines are shown in Figure 2.

An initial assessment was carried out to determine whether the isolated strains represented genetic novelty within the ISVV collection. To this end, the 94 original VNTR profiles were entered into the laboratory’s local database for comparison using Bionumerics software. Over the years, this database has been gradually enriched with profiles associated with approximately 11,000 clones recovered at the Bordeaux Wine Institute (ISVV), including most of the 50 commercial strains available on the global market. One of the initial objectives of our study was to supplement the collection with strains of grape varieties that had previously been underrepresented. The study focused in particular on Malbec, which was the most sampled grape variety in the experimental framework, with 7 wines out of 21. A total of 124 colonies were isolated from Malbec wines, representing 47 profiles, of which 79% (*n* = 37) were found to be novel and not represented in the database.

The analyses also revealed the presence of several profiles (n°35, 38, 41, 59, 66, 93) that were shared with commercial cultures. This result was unexpected since wines with spontaneous MLF were favored in the study in order to exclude any interference from microbial starters. However, commercial strains represent natural alleles associated with improved performance and were collected during successful spontaneous MLF. The presence of strains with similar or identical VNTR profiles in different wines and geographical areas is not unusual. Similarly, the persistence of genotypes characteristic of starters in cellars over several consecutive vintages has also been documented. It cannot therefore be ruled out that starters were used by partner wineries prior to 2021 (Reguant and Bordons, 2003; El Khoury et al., 2017).

Overall, a total of 44 VNTR profiles were orphan patterns (11.4%). The remaining 50 profiles were shared by various isolates, which were therefore clones of the same strain. A subset of 42 profiles had low frequency (encompassing 2–5 isolates), representing 31% of total isolates. In contrast, eight strains (with VNTR profiles n°13, 25, 38, 42, 45, 53, 61, 85) were abundant and were represented by more than 10 isolates (56.9% of all isolates). Profile 53 alone accounted for 26.6% of subtyped isolates (103 of 385 isolates) (Figure 2).

### 3.3 First insights in the intra-species diversity at the wine’s scale

The availability of 15–19 isolates for each sample provided a relative snapshot of the community’s dominant population. Alpha diversity metrics (richness and Shannon index) were examined (Figure 2). The main conclusion was that the sets of colonies associated with the different wines contained at least two cohabiting strains at the time of analysis, despite distinct selection conditions throughout the winemaking process. However, overall richness patterns were not uniform within wine samples or groups. Half of the wines (*n* = 11) had lower intraspecies diversity and were dominated by one strain, which accounted for more than 66% of all colonies collected. These dominant strains corresponded to profiles n°13 (W8), n°42 (W4), n°45 (W7), n°61 (W25) and n°53 (W6, W11, W12, W14, W17, W18, and W19) (Figure 2).

Conversely, the highest richness was observed in red wine W23, with 13 strains out of 18 colonies collected. Pearson correlation observed a statistically significant relationship between TPI content and diversity (*r* = 0.625). The highest diversity was observed in red wines with a higher TPI value (>50), which harbored 4 to 13 strains. These wines included Monastrell (W10) and Syrah (W2) wines from the G_D_ group, as well as most of the Malbec samples from the G_C_ group (Figure 1). In contrast, wines from the G_A_ and G_B_ groups contained 2 to 6 strains. These results are consistent with previous data from El Khoury et al. (2017), who found more *O. oeni* genotypes in red wines (1 to 11 strains, mean 4.23) than in white wines (1 to 4, mean 2.46). One hypothesis is that the presence of polyphenolic compounds positively modulates diversity, as has also been observed in other plant-based foods and in rumen ecosystems, since some of these compounds could be metabolized by bacteria and used as substrate. Moreover, these compounds may have an impact on the redox balance of the environment (Ntemiri et al., 2020; Jia et al., 2022). The Shannon index was not correlated with other wine-related factors (pH, ethanol content, bacterial population) (*r* < 0.5) or with the degree of MLF completion (Figure 1; Supplementary Table S1). In support of the latter observation, the number of VNTR profiles decreased only slightly after the end of MLF in sample W7, from five (W7_AF) to three (W7_DF) (Figure 2).

**Figure 3:**
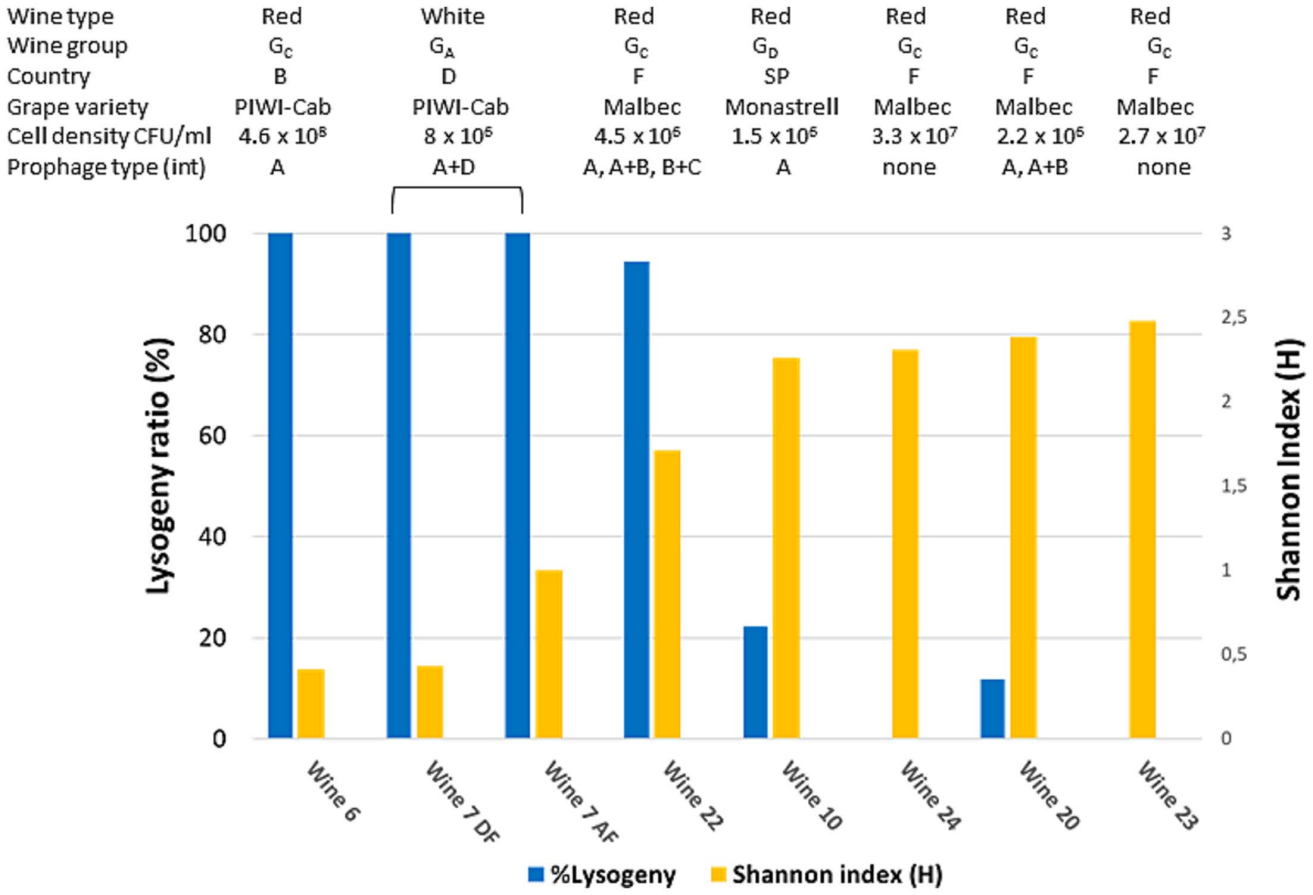
Frequency of lysogeny in sets of 15–19 colonies collected from seven wines. Wine characteristics (type, group, country, grape variety and cell density) are shown above the graph. Wine 7 was collected before (AF) and after completion of MLF (DF). All wine samples are ordered by increasing diversity (Shannon index) (yellow bars). Lysogeny was detected by multiplex PCR targeting the four genes coding for phage integrases (blue bars). The nature of the integrases (Int_A_ to Int_D_) and combinations thereof is also given. Piwi-Cab, Piwi-Cabernet.

In natural ecosystems, multiple lines of evidence suggest that diversity within complex bacterial populations is influenced by host-virus interactions (Voigt et al., 2021). For viruses infecting *O. oeni*, viral infection dynamics exist on a spectrum from highly lytic to lysogenic and/or chronic (Chaïb et al., 2022b). Temperate oenophages are particularly common, and more than half of the strains in the collections possess one to three prophages in their genome. Importantly, there is a consensus that a temperate phage transitions from lytic to lysogenic when its population grows faster as a prophage than as virions produced by lysis of infected cells, and vice versa for the transition from lysogenic to lytic (Roughgarden, 2024). Understanding this transition is currently a topic actively studied and discussed in other model ecosystems and accumulated data show that it strongly depends on the surrounding environmental conditions, including bacterial density and resource supply. All these data prompted us to analyze the distribution of prophages among the isolated strains according to wines and their diversity. To this end, a multiplex PCR assay was developed to simultaneously detect the four integrase genes (*int_A_* to *int_D_*) commonly associated with prophages in wine-associated *O. oeni* strains (Chaïb et al., 2022b) (Supplementary Table S2). The test was applied to all 15–19 colonies collected from seven red and rosé wines that underwent complete MLF and showed variations in richness. As shown in Figure 3, prophages were unevenly distributed in these wines, reflecting differences in the prevalence of lysogeny in the dominant population from one wine to another during MLF. As already identified in other natural ecosystems, an inversely proportional relationship appeared to exist between the frequency of lysogeny and infra-specific diversity within the tested subpopulations. Therefore, the process used to produce wines W6, W7, and W22 resulted in a less diverse population, where the dominant *O. oeni* strains were lysogenic. Conversely, the maintenance of several strains together in the population was associated with the absence of prophages in the genomes of co-resident strains. The differences between lysogeny rates in the sets of strains tested during MLF were independent of cell densities, which were high in the tested wines (ranging from 1.5 × 10^6^ to 4.6 × 10^8^ CFU/mL). No association was found with either the type of wine or any of the three chemical parameters measured in the samples. Among the data acquired, it is also worth mentioning sample W7, which is the only wine for which two kinetic points are available. No changes in lysogeny rate before and after the end of malic acid degradation were observed suggesting that the completion of MLF did not alter the trade-offs between the presumed fitness gain and the induction risk. However, slight differences were observed in the number and genotype of host strains before and after malic acid degradation (Figure 2), suggesting dynamic variations within the lysogen subpopulation in this wine during MLF. Our data need to be completed on a larger number of wines, with an emphasis on the kinetic monitoring of a larger number of parameters before and during MLF, such as the nature of the PC and temperature. Some factors yet to be identified are likely to affect the density and metabolic activity of bacterial communities, and probably have selective consequences for phages and their infection mechanism (Castledine and Buckling, 2024).

**Figure 4:**
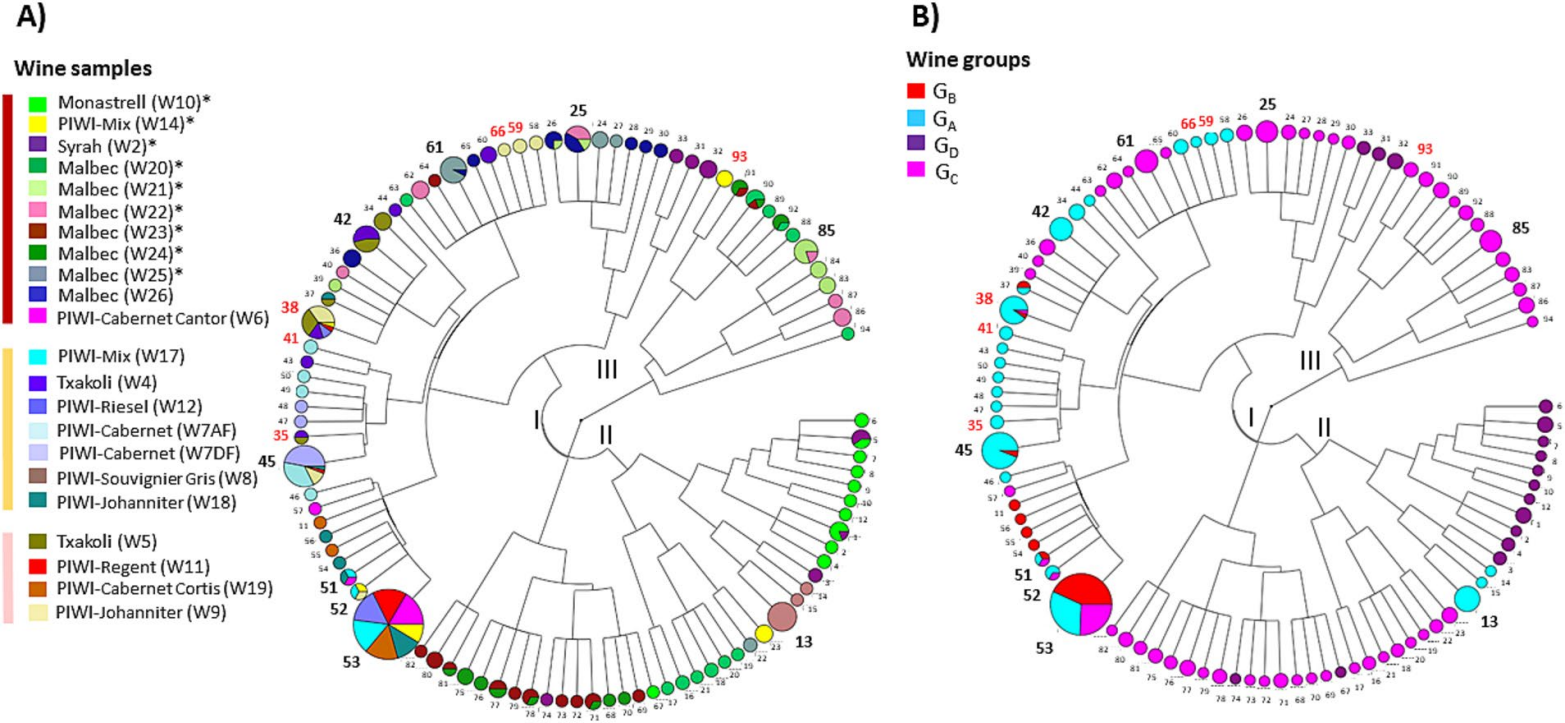
Cladograms representing the Inter-profile distance tree obtained with the 94 original VNTR profiles. **(A)** Distribution of profiles by wines. The area of the circles is proportional to the number of isolates belonging to the profile. More frequent profiles (n°) are represented in bold. Those shared with commercial cultures are in red. **(B)** Distribution of VNTR profiles by wine groups (G_A_ to G_D_).

Given that greater richness was observed in certain samples, the question arose whether genomic proximity was favored or not among the co-residents of the corresponding wines. To answer this question, phylogenetic trees were constructed from the different VNTR profiles (Figure 4). The analysis grouped the 94 profiles into two main groups, I and II, containing 60 and 22 profiles, respectively. A third minor group (III) of 12 profiles associated with red wines was also observed. In most samples, several isolates appeared as a single group, suggesting that the dominant populations at the time of collection in a given wine consisted of closely related strains. However, we observed significant inter-group differences for strains associated with eight red wines with high cell density and Shannon index, including W2, W10, W14, W21, W22, W23, W24, and W25 (Figure 4A). In these wines, co-resident isolates were distributed in two or three groups of VNTR profiles. Therefore, evident intra-sample diversity was observed in these wines, with higher richness and larger genomic distances between isolates.

Finally, the tree also revealed 21 cases of strain redundancy between wines (Figure 4A). However, in 16 cases, wines sharing the same strains belonged to the same wine group (Figure 4B). These included seven of the nine G_C_ red wines, and W2 and W10 (G_D_). Conversely, it is worth noting that the red wines grouped in the study into two distinct groups based on physicochemical differences (G_C_ and G_D_) did not share any common strains (Figure 1). Similarly, no common strains were identified between the three wines with an extreme value for any of the three monitored parameters (W10, ethanol; W5, low pH; and W24, TPI). Only a few strains were common to the different wine types (rosé, white, red). These included, in particular, frequent profiles n°38 and n°45 that were associated with wines from the G_A_ and G_B_ groups. Profile 53 was notable because it was associated with two wines of each type (white, red, and rosé) (Figures 2, 4B).

### 3.4 WGS assigns a set of representative isolates to two sublineages of phylogroup A

Based on VNTR analysis, a panel of 48 isolates was selected for WGS and comparative genomics. The full list is available in Supplementary Table S3. A first objective was to assess whether WGS could provide better strain typing resolution than VNTR. To this end, several clones sharing the same profile but collected from different wine samples/types (12 clones distributed in 5 VNTR profiles) were selected and sequenced. Relatedness was estimated using the number of single nucleotide polymorphisms (SNPs) separating isolates with the same profile. WGS confirmed that isolates with the same VNTR profiles had relatively low SNP variability. Pairwise SNP comparisons yielded between 0 and 22 SNPs (>85% of reads), respectively. It was concluded that the analyzed set of 12 isolates corresponded to 5 strains, reflecting the actual prediction accuracy by VNTR typing in *O. oeni*. In total, our study provides the complete genomes of 41 original strains of *O. oeni* accompanied by the chemical parameters of the wines from which the strains were isolated.

**Figure 5:**
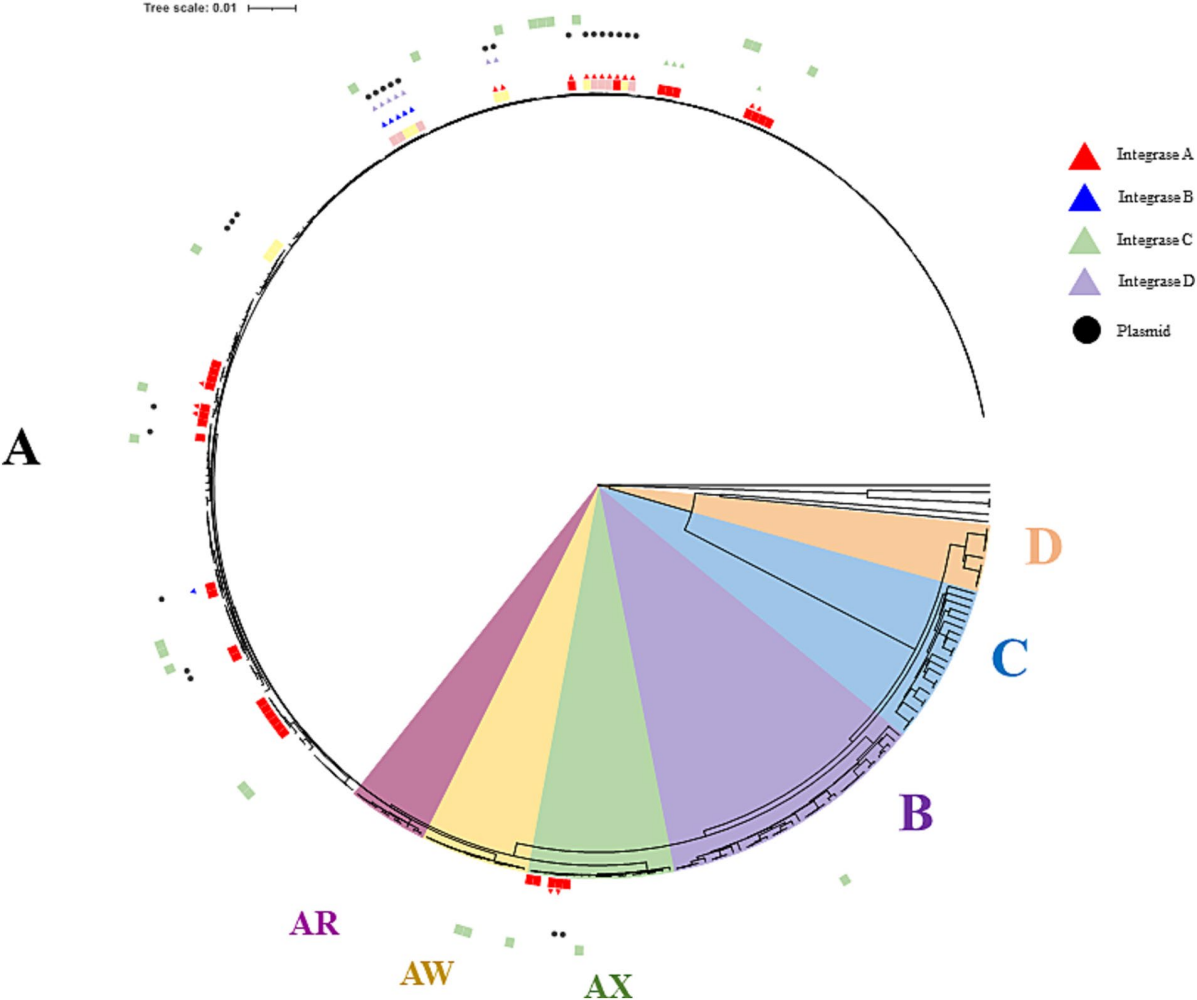
Phylogenetic tree of the relevant strains of *O. oeni* including the 48 sequenced isolates. Phylogroups D, C, and B are indicated in orange, blue and purple, respectively. The AX, AW and AR sub-lineages in phylogroup A are shown in burgundy, yellow and green, while other members are in white. The 48 isolates are distinguished on the outside of the tree with red, pink and yellow colors depending on the source (red, rosé and white wines, respectively). Currently available commercial strains are in green. Black dots and triangles represent plasmids and prophages, respectively. Int_A_, Int_B_, Int_C_, and Int_D_ prophages are in red, blue, green and purple, respectively.

A consequence of the variable stress combinations among the tested wines could be the selection of specific strain genotypes, providing the basis for adequate stress response mechanisms. Efforts were therefore made to include a few isolates from most of the wine samples (20 out of 21), including samples W5, W24, and W10, in which MLF occurred under the most extreme conditions of pH (pH 3.1), PC content (TPI 82), and ethanol (16.6%) in our set, respectively. The phylogenetic tree describing the relationships between the newly sequenced isolates and other publicly deposited high-quality genomes is shown in Figure 5. All sequenced *O. oeni* belonged to a single lineage of wine strains, previously described as phylogroup A. This is consistent with previous findings that strains from phylogroups B and C are rare in French wines. The latter are thought to be associated with the early stages of winemaking and are subsequently counter-selected during MLF (Balmaseda et al., 2023). So far, *O. oeni* strains from phylogroup A fall into a few subgroups, including the so-called AR and AW sublineages, formed by strains associated with red and white Burgundy wines (Lorentzen and Lucas, 2019). The newly isolated strains in our study do not belong to these groups, consistent with the distinct geographical origins of the 21 wines studied. It is important to note that the five W10 strains (Monastrell, G_D_ group) belonged to a more distant sub-lineage, previously called AX (Lorentzen et al., 2019). In the 5 years leading up to our study, the number of members in this group has slightly increased, reaching 15 members. The AX group strains were until now mainly associated with wines produced from red grape varieties (Malbec, Grenache, Pinot Noir and Cabernet Sauvignon) in various wine-producing countries (Lebanon, Australia, Argentina, France). This is the case of strain X2L, a potential MLF starter culture, with improved sensory characteristics in Argentine red wines (Mendoza et al., 2015). As already mentioned, the most striking characteristic observed for wine W10 is its high alcoholic strength (16.6%), medium TPI and rather permissive pH for bacterial growth (Figure 1). However, selection imposed by a similar combination of the three chemical factors in wine W2, which also belongs to the G_D_ group, but was fermented from Syrah grapes, did not result in the same genetic bases in the selected bacteria as those observed for the strains associated with W10. Specific selection agents led to distinct trajectories for the strains associated with AX, notably grape varieties, with specific abiotic (nature of PC, unidentified stressors) and biotic factors (such as phage pressure). Unfortunately, the deposited genome sequences available for other currently listed members of the AX sub-lineage did not provide a better understanding of the chemical composition of the original wines fermented by these strains. Therefore, these partial characterizations in databases currently represent a major obstacle to comparisons and the identification of oenological characteristics that could select for these unique genomic profiles gathered in the AX sub-lineage.

### 3.5 WGS provide novel insights in the mobilome of *Oenococcus oeni*

**Figure 6:**
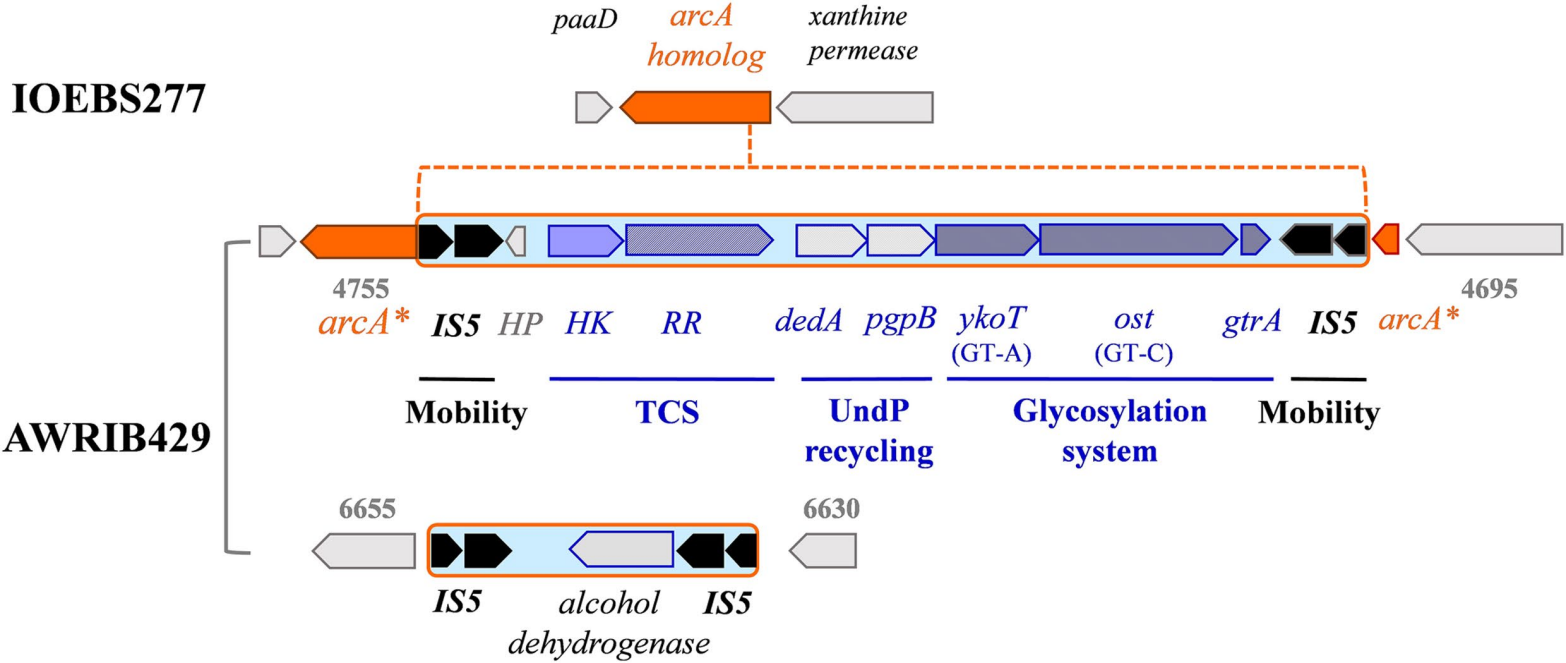
Representation of the IS configurations surrounding the putative glycosylation cluster and alcohol deshydrogenase gene in *O. oeni* IOEBS277 and AWRIB429. Gene numberings refer to locus tag J3U91_RS0 (strain AWRIB429).

**Figure 7:**
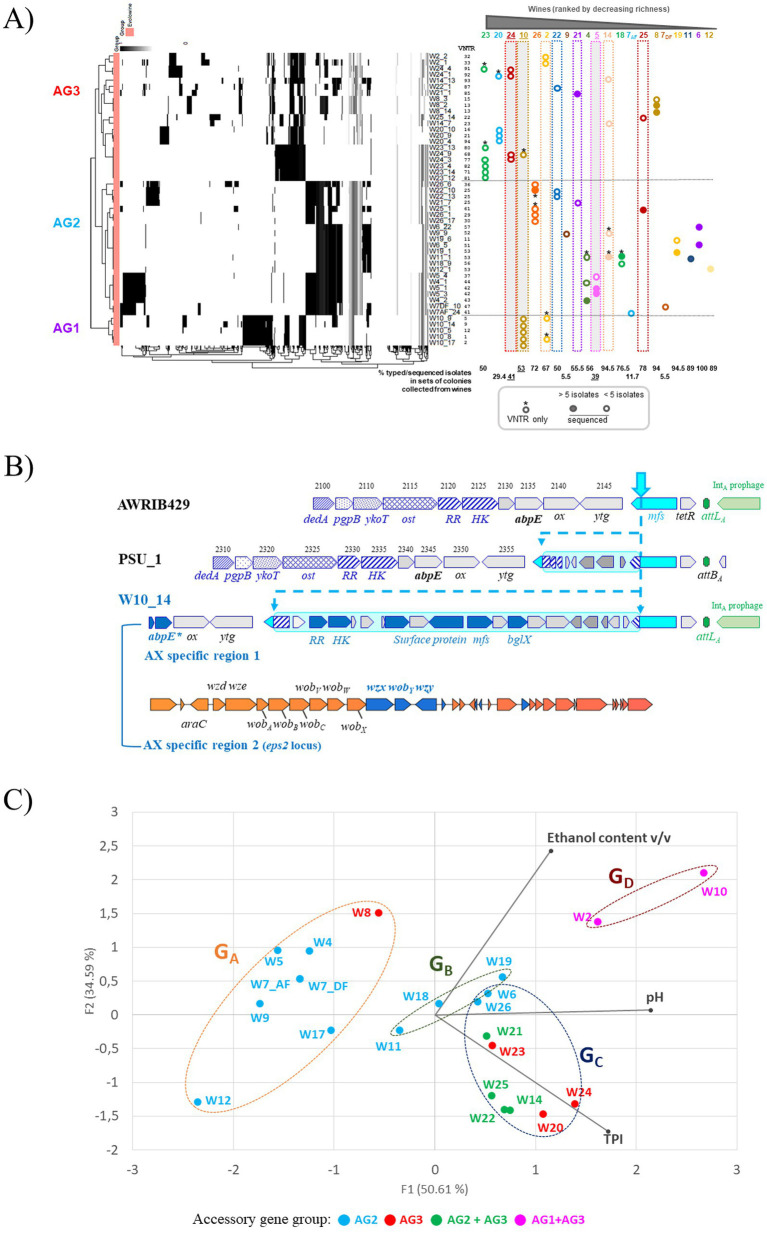
Intraspecific diversity in *O. oeni* and genetic variations between co-resident strains in each of the 21 wines analyzed. **(A)** The left panel shows a heat map representing the hierarchical grouping of the absence/presence matrix of the different accessory genes (x axes) annotated in the different preliminary genomes (y axes); the three clusters observed are named AG1, AG2, and AG3; PPanGGOLiN was used with default settings, including 80% coverage. The right panel summarizes the available data (VNTR, WGS) for co-resident strains in the 21 wines and their membership in the AG clusters. The three wines with an extreme value for any of the three monitored parameters (W10, ethanol; W5, low pH; and W24, TPI) are shaded in gray. For each wine, the percentage of isolates characterized in the corresponding set of colonies collected is also presented below the graph; **(B)** Specific genes found in members of the AX sub-group, represented by strain W10_14. All AX-specific genes in regions 1 and 2 are in blue. Region 1 (boxed in cyan blue) is integrated in a gene specifying an MFS transporter upstream of the *attB*_A_ site. The locus displayed multiple deletions in strain PSU-1. Locus tags were J3U91_RS0, and OEOE_RS0 for strains AWRIB429 and PSU-1, respectively. **(C)** genetic proximity between strains identified in wines from groups G_A_, G_B_, G_C_, and G_D_; colors represent the strains’ membership in one or more accessory genome clusters.

HGT events are thought to be frequent in *O. oeni* and the loss of CRISPR-Cas would facilitate them (Barchi et al., 2022). This prompted us to monitor the presence of MGEs in whole genome assemblies of all isolates selected from various wines. In this global inventory, we particularly looked for novel structures and/or associations of mobile entities not described in the current literature. Our analysis of the predicted mobilome identified 4 to 113 nomadic genes tracked by isotopes, transposons, or plasmids in each genome (Supplementary Figure S2). The main features are presented below and illustrated in Figures 5–7.

A total of 149 ISs belonging to six families were annotated by ISFinder. Consistent with previous results, the IS*30* family was the most abundant and widespread group (*n* = 141), with members associated with all strains (El Gharniti et al., 2012). Other IS elements belonged to the IS*L3* (*n* = 52), IS*3* (*n* = 47), IS*L1* (*n* = 5), IS*5* (*n* = 2), and IS*6* (*n* = 2) families. IS copy numbers varied considerably among strains. The presence of an atypical member of the IS5 family, consisting of two consecutive genes encoding a transposase, is new information. The deduced amino acid sequences showed 59 and 64% identity, respectively, with the putative transposases named ISCARN82.aa1 and ISCARN82.aa2, obtained from metagenomic data of soil samples. Examination of the nucleotide sequences of the two ORFs in *O. oeni* supports the hypothesis of a frameshift, as previously reported for LAB transposases (Nicoloff and Bringel, 2003). More interestingly, the inferred IS*5* sequences appeared twice in a given genetic neighborhood in some of the newly isolated strains (Figure 6). This may be the signature for IS*5*-like composite transposons in *O. oeni*. A first structure was frequent among strains, except those associated to W20 (Figure 6). It was composed of an alcohol dehydrogenase gene, bounded by inversely-oriented copies of IS*5* and the region has been earlier identified as a MGE (Margalef-Català et al., 2017). A second, more complex putative transposon, IS*5*-type, with eight cargo genes, was common to 22 of the sequenced isolates and was also detected in several commercial strains (Onetto et al., 2021). This transposon-like structure interrupts a gene specifying a protein 65% identical to ArcA arginine deaminase (Figure 6). The targeted gene was preceded by an ORF possibly dedicated to ribonucleotide synthesis (xanthine permease). The downstream *pad* and *suf* genes are associated with phenylacetate catabolism and Fe-S cluster assembly during iron deficiency, respectively (Corless et al., 2020). Based on the homology and predicted topology of the eight transposon-associated proteins, we suggest that the putative mobile element may specify a putative three-gene glycosylation system, involved in the decoration of *O. oeni* cell wall glycopolymers (Theodorou et al., 2021; Maes et al., 2025). Indeed, our analyses predict the presence of a GT-A fold family 2 (YkoT-like) glycosyltransferase (99.94% probability; *e*-value 1.4 e-22 with GtrB from *Synechocystis* sp., 5EKE_C). The protein would load an undecaprenol lipid transporter (C55-P, also known as UndP) with a sugar from a nucleotide-diphosphate-sugar donor to form a membrane-associated Und-P-sugar. Then, the Und-P-sugar would be transported to the outer leaflet of the cell membrane by a small integral membrane flippase usually possessing two C-terminal transmembrane α-helices (GtrA) (98.46% probability; *e*-value 1.9 e-6 with dolichyl phosphate mannose synthase from *Pyrococcus furiosus*). The locus also encodes a GT-C family glycosyltransferase (Pmt2-like) with 13 transmembrane helices and an active-site aspartate-containing motif in the first extra cytoplasmic loop (99.82% probability; *e*-value 4.6 e-17 with ArnT from *Cupriavidus metallidurans*). This Gtf (Ost, for oligosaccharyltransferase) is a good candidate enzyme for transferring the glycosyl group from the lipid intermediate to an undefined acceptor substrate on the outside of the cell. Finally, adjacent to these genes were two ORFs specifying a putative recycling system for UndP, consisting of a Pap2-phophatase and a lipid carrier of the DedA COG 5228 family (Todor et al., 2023). The putative glycosylation-related cassette was located downstream of a putative two-component system (TCS) of the OmpR/PhoB family. The DNA-binding response regulator protein (RR) resembles the OmpR-like protein MtrA associated to the MtrAB system in *Mycobacterium tuberculosis* (probability 99.94%; *e*-value 1.4 e-22). In the latter model, the TCS tunes cell division in specific environments and the whole regulon harbors 71 genes (Peterson et al., 2023). Transposons carrying TCS were also recently shown to use a similar mechanism to control gene expression of antibiotic resistance determinants in Enterococci (Udaondo et al., 2022). The TCS identified in *O. oeni* may represent a mechanism for transducing signals across membranes and could be involved in the glycosylation of a cell-envelope moiety in response to abiotic and/or biotic constraints.

Non-conjugative plasmids identified as circular contigs were identified in 50% of isolates and associated with 16 wines (Figure 5). This high frequency could result from the initial decision to limit the number of passages during the bacterial isolation steps, to avoid loss and/or genomic rearrangements of MGEs. The cryptic and rolling circle replicating plasmids pRS2 and pRS3, previously characterized by Mesas et al. (2001), were rare and limited to three strains. Slightly modified versions of the theta replicating plasmid pOENI-1 (18.3 kb) and its larger version named pOENI-1v2 (21.9 kb) (Favier et al., 2012) were detected and the latter had a higher occurrence. The pOENI-1 derivatives of strain W24_1 contained the IS30, IS6 and IS3 elements. The latter could decrease the fitness cost of the plasmid by disrupting genes harmful to the host, as recently proposed by Wedel et al. (2023). A larger plasmid was detected in strain W10_14 and was named pOENI-2 (Supplementary Figure S3). Its distinguishing feature was the presence of genes related to xylose metabolism (non-PTS transporter, xylose isomerase, xylulokinase). A *xylR* gene was also present and corresponds to a transcriptional repressor of the ROK family of the xylose operon. Xylose is present in wine at low concentrations at the end of alcoholic fermentation and the ability to grow on this sugar is variable depending on the strains (Cibrario et al., 2016). A gene specifying a putative auxin efflux transporter (AEC) was found upstream of the putative *xyl* operon. The protein shows 25% identity (*e*-value 4 × 10–12) with that of *Bacillus licheniformis* AEC which has recently been implicated in the efflux of indole-3-acetic acid, an auxin phytohormone (Rai et al., 2021).

In addition to harboring transposons and plasmids, oenococci are also susceptible to infection by genetically diverse temperate phages. A total of 34 full-length prophages were predicted across all sequenced bacterial isolates. The percentage of lysogeny was 58.3% (26 out of 48 isolates), which is consistent with previous data (Claisse et al., 2021; Chaïb et al., 2022b). Lysogens were associated with 16 of 21 wines of all types. The prophages demonstrated well-conserved patterns in genome organization and synteny compared to known viral genomes in the species, and 30 types were identified. Analysis of phage type distribution revealed that the majority of isolates carried the phage types Int_A_ (17), Int_B_ (6), Int_C_ (4), and Int_D_ (7) (Chaïb et al., 2022b) (Figure 5). Consistent with previous data, prophages belonging to the Int_A_, Int_C_, and Int_D_ categories were integrated at a single location in the oenococcal genome (*attB*_A_, *attB*_C_, and *attB*_D,_ respectively). In contrast, Int_B_ prophages were inserted either at the *attB_B_* site or the *attB*_F_ site, as previously reported (Claisse et al., 2021). Polylysogenic strains represented 31% of the lysogenic strains (n = 8) in the study, and all harbored two prophages. In three cases, Int_A_ prophages were associated with Int_D_ or Int_C_ members, and this is a frequent association in the species (Claisse et al., 2021). More surprisingly, the remaining five polylysogens harbored a novel Int_B_-Int_D_ combination. Another intriguing feature, so far unresolved, is that Int_B_ prophages targeted the *attB*_F_ site in polylysogens, while the *attB*_B_ site was used in the single monolysogen isolated in the study.

Finally, we observed that a total of 24 of the 26 lysogens also harbored a plasmid (Figure 5). The lack of obvious intragenomic conflict between these MGEs suggests that cross-regulation occurs between them. Stabilization of prophages by plasmids has recently been demonstrated in other genera such as Roseobacter (Tuttle et al., 2022). In *O. oeni*, cross-regulation might involve toxin-antitoxin systems, which are commonly found on MGEs in this species (Favier et al., 2012; Claisse et al., 2021). The coexistence of homologous and non-homologous systems on MGEs in *O. oeni* represents a topic that deserves further exploration in the wine laboratory setting.

### 3.6 Search for signature of selection in accessory genomes

A few studies have suggested that genetic plasticity may allow *O. oeni* strains to acquire genetic determinants to resist oenological stresses (Bon et al., 2009; Margalef-Català et al., 2017; Chi et al., 2025). To further document this hypothesis, the pangenome of the 48 accessions was calculated with 400 other publicly deposited genomes using PPanGGOLiN (Bazin et al., 2020; Gautreau et al., 2020). After extracting persistent genome sequences, the remaining sequences were designated as the accessory genome. It consisted of various regions of genomic plasticity (gene clusters consisting of shell and cloud genomes in the pangenome architecture) (Supplementary Figure S2). All newly sequenced isolates are distributed into three distinct patterns, named AG1 (*n* = 5), AG2 (*n* = 22), and AG3 (*n* = 21) (Figure 7A). Next, the specific features of the three AG profiles were analyzed with the objective to identify elements which may explain the ability of strains to persist in a particular wine type and/or wine composition. It was observed that the few sequenced isolates associated with wines with the most extreme individual parameters, namely W10 (16.6% ethanol), W5 (pH 3.1) and W24 (TPI 82), belonged to AG1, AG2, and AG3, respectively. The situation was more complex for isolates from other types or wine groups, which did not always appear as single AG clusters. However, there was an overlap between the most acidic white and rosé wines from G_A_ and G_B_ groups and the AG2 profile, with one exception. We also observed that the AG3 pattern grouped the sequenced isolates from the red wines with higher TPI and/or ethanol content in the sampled wines (Figure 7A).

The AG1 profiles grouped the five W10 strains, organized into the AX lineage. The genomic subpopulation sharing the AG1 profile showed important differences, with significant variations in the presence and absence of genes, making it a potentially important factor in the phenotypic characteristics of the corresponding strains. Most genes were associated with specific regions of plasticity, with no obvious traces of MGE. An in-house program identified 16 specific putative protein sequences (99–100% identity) conserved among the five W10-associated strains and other known members of the AX sub-lineage. The AX-specific sequences included three small proteins (67–123 aa) corresponding to the truncated N-terminus of a putative lipid permease (MbsA), an aggregation factor protein, and an ApbE-like modifying enzyme involved in flavoprotein maturation and extracytosolic redox (Huang et al., 2024). The presence of one or two mutations in the corresponding ORFs was specific to the AX lineage. The scope and importance of ApbE-type flavinylation in microbial energy metabolism are emerging. They may play important roles in various mechanisms of microbial energy metabolism and be involved in the function of both respiratory and non-respiratory reductases (Light et al., 2019). The three truncated genes were located in plastic genomic regions immediately upstream of the *attB*_D_, *attB*_C_, and *attB*_A_ sites, respectively, which are used for site-specific integration of prophages in *O. oeni* (Claisse et al., 2021). The region inserted upstream of the *attB*_A_ site mentioned above was highly plastic. In addition to the presence of a deleted *apbE* gene, region 1 also contains six AX-specific genes, which are absent or mutated in strains with AG2 and AG3 profiles (Figure 7B). Region 1 was found to be integrated into a gene specifying an MFS transporter, resulting in the presence of two hybrid genes at the borders (Figure 7B). It specified a putative extracellular β-glucosidase, a surface protein, a two-component system, and an MFS transporter. It is worth mentioning that a second island resembling the aforementioned glycosylation locus (see Figure 6) was also found upstream of region 1 and was not specific for the AG1 profile (Figure 7B). Comparisons of the two putative glycosylation loci highlighted differences in gene content and order, and the absence of evidence upstream of region 1 of the IS sequences and the putative *gtrA* flippase gene. Pairwise comparisons were performed for the YkoT, Ost, and DedA protein sequences and showed identities of 52, 23, and 43%, respectively, suggesting distinct origins for the two loci. Also observed in Figure 7B, the remaining seven AX-specific proteins were associated with the *eps2* locus, which is responsible for the production of capsular exopolysaccharides (EPS) in *O. oeni* (Dimopoulou and Dols-Lafargue, 2021; Maes et al., 2025). The region is highly variable in the species and a total of 15 distinct patterns of the *eps2* cluster have been reported so far, eight of which were associated with members of phylogroup A (Dimopoulou et al., 2014). An interesting finding is that all members of the AX sub-lineage displayed the same architecture of the *eps2* locus, suggesting their ability to produce a specific capsule compared to other phylogroup A strains. Such a capsule may increase cell survival under specific winemaking conditions, such as those associated with W10 (Nucci et al., 2022).

Conversely, genomes associated with cluster AG2 appear to have more functional mobile elements, with the ability to be horizontally transferred, while those with AG1 and AG3 may be enriched with remnants of mobile elements that can no longer be mobilized (Figure 7A). First, prophage distribution explained some differences because the occupation of the *attB*_C_, *attB*_D_, and *attB*_F_ sites by a prophage was a common and exclusive trait shared by strains with the AG2 pattern. Conversely, Int_A_ prophages at the *attB*_A_ site were distributed uniformly in strains from all three clusters. It was also observed that the four related lysogens containing an Int_B_-Int_D_ combination belonged to cluster AG2. They were associated with white (W4) and rosé (W5) Txacoli wines collected from the same winery. The failure to detect this combination of Int_B_-Int_D_ prophages in previous *in silico* inventories may be due to the instability of MGE caused by repeated subcultures and/or specific winemaking conditions. It would now be advisable to isolate new strains from Txacoli wines which are currently underrepresented in databanks. Second, the AG2 model included a higher representation of strains with pOeni plasmids (73%, 16 out of 22 isolates), while values of 20 and 24% were observed for strains in AG1 and AG3 clusters, respectively. Third, all strains with AG2-like accessory genomes harbored the IS*5*-transposon carrying the putative glycosylation system and two-component system HK–RR signaling pathway (100% in strains from AG2 and absent from AG1 and AG3) (Figure 6). If it turns out that the transposon is indeed involved in the envelope modification, this may interfere with specific phage/host interactions, as shown in *Lactococcus lactis* (Theodorou et al., 2021) and/or help strains cope with specific wine-related stresses. Importantly, the role of the transposon-associated TCS has been recently investigated by cloning the corresponding genes from *O. oeni* SD2a into *Lactiplantibacillus plantarum* (Zheng et al., 2024). The authors demonstrated that TCS controlled the expression of several genes in the recombinant strain. Overall, the presence of the system increased ATP consumption and improved the fluidity, permeability, and cell membrane integrity of early log-stage LAB, thus improving its overall tolerance to acid stress. Consistent with these data, most of the sequenced isolates from white and rosé wines at the most acidic pH in this study have an AG2 profile and carry the TCS on the transposon. The exceptions were the three strains derived from W8, a white wine with a higher ethanol content (14%) and pH, which had a profile similar to that of AG3. However, within the AG2 profile, the presence of the transposon was not exclusively associated with white and rosé wine strains. It was also detected in five red wine strains (W6, W21, W22, W25, and W26) that did not have a low pH (Figure 7A). The input detected by the putative TCS is currently unknown (Zheng et al., 2024). It may not be acidity *per se*, but rather a signal common to wines exhibiting the AG2 pattern.

Another important question is whether microdiversity may be also required to help to maintain the stability and functioning of the *O. oeni* population. We therefore reanalyzed the genetic profiles within the groups of dominant strains associated with the same wine in order to assess their genetic proximity. These preliminary data suggest that different selective pressures led to the formation of groups of genetically related individuals, particularly in white and rosé wines with moderate ethanol content. However, cohabiting strains with contrasting genetic profiles were also observed in 7 wines, including 6 red wines (Figure 7A). The latter were characterized by the maintenance of genetically different strains, with mixed AG3 + AG1 (W2 and W10) or AG3 + AG2 profiles (W14, W21, W22, and W25) (Figure 7C). No wines harbored strains with AG1 and AG2 profiles together, which may suggest incompatibility groups (Figure 7C). The phenomenon of within-sample infra-species genetic diversity has been recently reported in fermented milk ecosystems (You et al., 2023). How such an infra-species genome heterogeneity is generated and maintained in the wine microbiota remains to be answered. Social diversification should be investigated. Interestingly, the genetic basis of sociability could be identified *in silico* in *Leuc. mesenteroides* using the SOCfinder tool showing that the estimated proportion of the genes that are categorized as cooperative is 1.5% (Belcher et al., 2023).

## 4 Conclusion

Our data show that the selection that occurs within the *O. oeni* population during fermentation nevertheless preserves a certain level of intraspecific diversity in wines, characterized by the coexistence of several strains with similar or distinct genetic profiles. At this stage, it remains difficult to link the homogeneous/heterogeneous nature of the profiles to the types of wines or to the three chemical parameters (ethanol, pH, and TPI) monitored. Technical advances in sequencing and bioinformatics analyses based on whole genome sequencing (WGS) will enable us to better respond to these new challenges of intraspecific diversity in the near future, and assess whether this may increase fitness through diversification of strategies or division of labor in specific production environments. One avenue that can be explored immediately is to study in detail the transposon carrying a two-component system upstream of genes dedicated to glycosylation in *O. oeni* as this may provide valuable insights into the potential performance of strains and aid in the development of commercial starters for winemaking applications.

## Data Availability

The datasets presented in this study can be found in online repositories. The names of the repository/repositories and accession number(s) can be found in Supplementary Table S3.
