# Supplementary figures and images for: A reassessment of the infra-species diversity patterns in the wine-associated Oenococcus oeni
Source: Front Microbiol. 2025 Sep 24;16:1657712. doi: 10.3389/fmicb.2025.1657712 (PMC12504390; doi:10.3389/fmicb.2025.1657712)

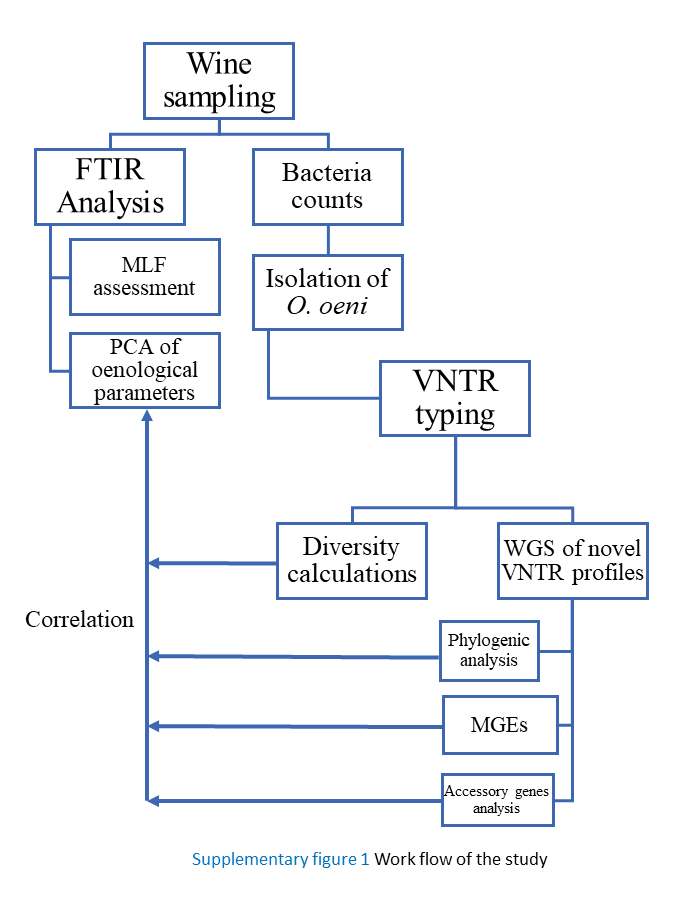

Supplement: Supplementary file 1 [file Image_1.TIF]

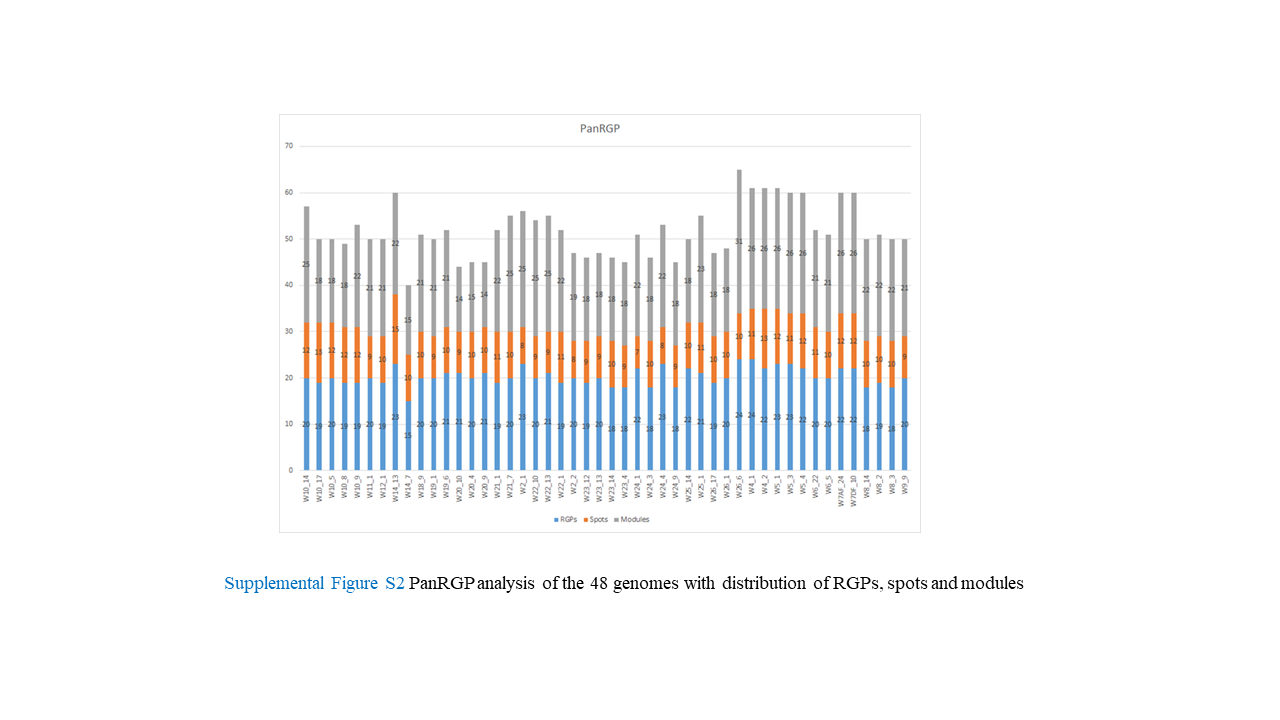

Supplement: Supplementary file 2 [file Image_2.TIF]

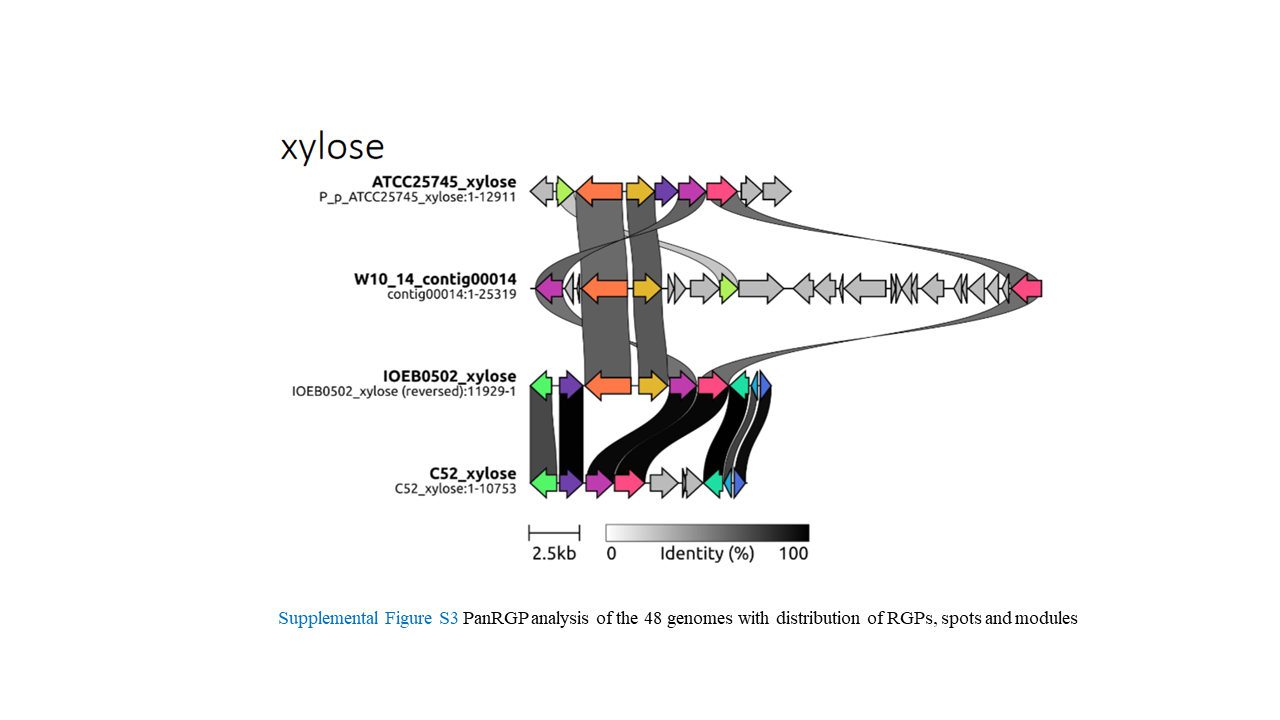

Supplement: Supplementary file 3 [file Image_3.TIF]
