# Supplementary material for: A reassessment of the infra-species diversity patterns in the wine-associated Oenococcus oeni
Source: Front Microbiol. 2025 Sep 24;16:1657712. doi: 10.3389/fmicb.2025.1657712 (PMC12504390; doi:10.3389/fmicb.2025.1657712)
Supplement: Supplementary file 4 [file Table_1.DOCX]

**Supplemental Table S1** LAB counts, physical-characteristics and selected VNTR profiles for whole sequencing associated to 22 samples from 21 wines.

| **Wine & country** | | **Type** | **Wine group** | **LAB**  **CFU/mL** | **Cepage** | **pH** | **TAV (%)** | **Malic acid**  **(g/L)** | **Lactic acid**  **(g/L)** | **TPI** |
| --- | --- | --- | --- | --- | --- | --- | --- | --- | --- | --- |
| 5 | SP | Rosé | A | 3.3 x 10^2^ | Txakoli | 3.1 | 13.19 | 0 | 1.98 | 3 |
| 9 | NL |  |  | 8.8 x 10^6^ | PIWI-Johanniter | 3.34 | 12.17 | 0 | 3.03 | 10 |
| 11* | B |  | B | 3.3 x 10^7^ | PIWI-Regent Rosé | 3.75 | 11.45 | 2.54 | 0.56 | 10 |
| 19* | NL |  |  | 2.2 x 10^3^ | PIWI-Cabernet Cortis | 4.01 | 12.72 | 3.42 | 0.28 | 19 |
| 18* |  | White |  | 2.8 x 10^1^ | PIWI-Johanniter | 3.81 | 12.15 | 4.19 | 0.13 | 11 |
| 17 |  |  | A | 1.5 x 10^6^ | PIWI-Mix | 3.64 | 11.55 | 0 | 2.6 | 12 |
| 12 | B |  |  | 1.5 x 10^7^ | PIWI-Riesel | 3.28 | 9.71 | 0.05 | 2.33 | 7 |
| 4 | SP |  |  | 7.6 x 10^6^ | Txakoli | 3.38 | 13.2 | 0.23 | 1.26 | 4 |
| 7_AF_* | D |  |  | 2.7 x 10^2^ | PIWI-Cabernet Blanc | 3.25 | 12.56 | 4.09 | 0 | 5 |
| 7_DF_ |  |  |  | 8 x 10^6^ |  | 3.42 | 12.56 | 0 | 2.3 | 5 |
| 8 |  |  |  | 3.6 x 10^6^ | PIWI-Souvignier Gris | 3.58 | 14.07 | 0.21 | 1.67 | 4 |
| 20 | F | Red | C | 2.2 x 10^6^ | Malbec | 3.83 | 11.44 | 0.06 | 1.71 | 67 |
| 21 |  |  |  | 1.1 x 10^6^ |  | 3.54 | 12.87 | 0.22 | 2.09 | 54 |
| 22 |  |  |  | 4.5 x 10^6^ |  | 3.56 | 11.82 | 0.25 | 1.36 | 74 |
| 23 |  |  |  | 2.7 x 10^7^ |  | 3.55 | 12.75 | 0.31 | 1.93 | 57 |
| 24 |  |  |  | 3.3 x 10^7^ |  | 3.73 | 12.21 | 0.19 | 2 | 82 |
| 25 |  |  |  | 3.3 x 10^7^ |  | 3.71 | 11.5 | 0.08 | 2.14 | 56 |
| 26* |  |  |  | 3.4 x 10^6^ |  | 3.67 | 12.98 | 0.67 | 1.52 | 34 |
| 14* | B |  |  | 7.6 x 10^6^ | PIWI-Mix | 3.72 | 11.44 | 3.51 | 0.24 | 64 |
| 6 |  |  |  | 4.6 x 10^8^ | Piwi-Cabernet Cantor | 3.74 | 13.56 | 0.45 | 1.27 | 34 |
| 10* | SP |  | D | 1.5 x 10^6^ | Monastrell | 4.02 | 16.56 | 1.21 | 0.43 | 50 |
| 2 |  |  |  | 2.5 x 10^7^ | Syrah | 4.14 | 14.29 | 0.06 | 0.96 | 51 |

TAV, AF and DF represent sample W7, before and after completion of MLF. Asterisks represent uncomplete or stuck MLF. Wines with stuck or uncomplete MLF are indicated with an asterisk and residual malic acid concentration is shaded in grey. SP, Spain ; NL, The Netherlands ; B, Belgium ; D, Germany ; F, France.

**Supplemental Table S2** Primers used in this study

| **Target gene** | **Primer** | **Reference strain used to design primers and 5’-3’ sequence** | |
| --- | --- | --- | --- |
| *intA* | intE mf | IOEBS277 | CAGCAATAAAGGAAAGTCAGCAG |
|  | intA mr |  | TCCGACATTACAAGGGCACT |
| *intB* | intB mf |  | GCGCGTTTCTGGTTTATTGGT |
|  | intB mr |  | TCTTCGTTTTCCCTGAGCGT |
| *intC* | intC mf |  | CTATACACACTGGCATGCGT |
|  | intC mr |  | TGTCTTCTTCGTTTTCCCGG |
| *intD* | intD F |  | CGGAAAATATTATCAAGCACGAG |
|  | intD R |  | TTCAGCGTGATCTTTACCAAAAT |

**Supplemental Table S3** VNTR profiles and occurrence of 94 isolates in 21 wines and list of accession numbers of 48 sequenced isolates

| Wine type and number | | Set of colonies analyzed | | Sequenced strains | | | |
| --- | --- | --- | --- | --- | --- | --- | --- |
|  |  | Nb | VNTR profiles and occurrence  in the wine sample | Name | VNTR profile | Accession number | F (%) |
| White | W11 | 18 | **53** (x16), 38 (x1), 45 (x1) | W11_1 | 53 | JBHNXZ000000000 | 89 |
|  | W19 | 18 | **53** (x16), **11** (x1), 55 (x1) | W19_1 | 53 | JBHNXU000000000 | 94.5 |
|  |  |  |  | W19_6 | 11 | JBHNXT000000000 |  |
|  | W5 | 18 | 38 (x6), **42** (x6), 34 (x4), 35 (x1), **37** (x1) | W5_1 | 42 | JBHNWT000000000 | 39 |
|  |  |  |  | W5_3 | 42 | JBHNWS000000000 |  |
|  |  |  |  | W5_4 | 37 | JBHNWR000000000 |  |
|  | W9 | 18 | 38 (x7), 45 (x4), 58 (x2), 66 (x1), 59 (x2), **52** (x1), | W9_9 | 52 | JBHNWJ000000000 | 5.5 |
| Rosé | W12 | 18 | **53** (x16), 38 (x2) | W12_1 | 53 | JBHNXY000000000 | 89 |
|  | W18 | 17 | **53** (x12), 37 (x1), 45 (x1), 51 (x1), 54 (x1), **56** (x1), | W18_9 | 56 | JBHNXV000000000 | 76.5 |
|  | W4 | 16 | **42** (x7), 60 (x3), 35 (x1), 38 (x3), **43** (x1), **44** (x1) | W4_1 | 44 | JBHNWV000000000 | 56 |
|  |  |  |  | W4_2 | 43 | JBHNWU000000000 |  |
|  | W7 | 17 | 45 (x12), **41** (x2), 46 (x1), 49 (x1), 50 (x1) | W7AF_24 | 41 | JBHNWO000000000 | 11.7 |
|  |  | 18 | 45 (x16), **47** (x1),48 (x1), | W7DF_10 | 47 | JBHNWN000000000 | 5.5 |
|  | W8 | 17 | **13** (x15), 14 (1), **15** (x1) | W8_2 | 13 | JBHNWL000000000 | 94 |
|  |  |  |  | W8_3 | 15 | JBHNWK000000000 |  |
|  |  |  |  | W8_14 | 13 | JBHNWM000000000 |  |
|  | W17 | 18 | **53** (x16), **52** (x1), 51 (x1) | none | - | - | 94.5 |
| Red | W10 | 17 | **1** (x4), 4 (x2), **5** (x2), 6 (x2), **2** (x1), 7 (x1), 8 (x1),  **9** (x1), 10 (x1), **12** (x1), 67 (x1) | W10_14 | 9 | JBHNYE000000000 | 53 |
|  |  |  |  | W10_17 | 2 | JBHNYD000000000 |  |
|  |  |  |  | W10_8 | 1 | JBHNYB000000000 |  |
|  |  |  |  | W10_9 | 5 | JBHNYA000000000 |  |
|  |  |  |  | W10_5 | 12 | JBHNYC000000000 |  |
|  | W2 | 15 | **32** (x4), **33** (x2), 3 (x2), **5** (x3), 31 (x2), **1** (x1), 74 (x1) | W2_1 | 33 | JBHNXS000000000 | 67 |
|  |  |  |  | W2_2 | 32 | JBHNXR000000000 |  |
|  | W20 | 17 | (90x3), (88x2), (18x2), (**16**x2), (**94**x1), (**92**x1), (89x1), (63x1), (**21**x1), (20x1), (19x1), (17x1), | W20_10 | 16 | JBHNXQ000000000 | 29.4 |
|  |  |  |  | W20_4 | 94 | JBHNXP000000000 |  |
|  |  |  |  | W20_9 | 21 | JBHNXO000000000 |  |
|  | W14 | 18 | **53** (x9), **23** (x4), **93** (x3), **52**(x1), 38 (x1) | W14_13 | 93 | JBHNXX000000000 | 94.5 |
|  |  |  |  | W14_7 | 23 | JBHNXW000000000 |  |
|  | W21 | 18 | **85** (x8), 83 (x3), 84 (x3), **25** (x2), 26 (x1),  39 (x1) | W21_1 | 85 | JBHNXN000000000 | 55.5 |
|  |  |  |  | W21_7 | 25 | JBHNXM000000000 |  |
|  | W22 | 18 | **25** (x5), 62 (x4), 86 (x4), **85** (x2), **87** (x2),  40 (x1) | W22_1 | 87 | JBHNXL000000000 | 50 |
|  |  |  |  | W22_10 | 25 | JBHNXK000000000 |  |
|  |  |  |  | W22_13 | 85 | JBHNXJ000000000 |  |
|  | W23 | 18 | **80**^b^(x3), **71** (x2), **77**^b^ (x2), 78 (x2), 64 (x1), 69 (x1), 72 (x1), 73 (x1), 79 (x1), **81** (x1), **82** (x1), 90 (x1), 91 (x1) | W23_12 | 81 | JBHNXI000000000 | 50 |
|  |  |  |  | W23_14 | 71 | JBHNXG000000000 |  |
|  |  |  |  | W23_13 | 80 | JBHNXH000000000 |  |
|  |  |  |  | W23_4 | 82 | JBHNXF000000000 |  |
|  | W24 | 17 | 75 (x3), **92** (x2), 76 (x2), **77** (x2), **68** (x1), 70 (x1),  71 (x1), 78 (x1), 81 (x1), 90 (x1), **91** (x2) | W24_1 | 92 | JBHNXE000000000 | 41 |
|  |  |  |  | W24_3 | 77 | JBHNXD000000000 |  |
|  |  |  |  | W24_9 | 68 | JBHNXB000000000 |  |
|  |  |  |  | W24_4 | 91 | JBHNXC000000000 |  |
|  | W25 | 18 | **61** (x12), 24 (x3), **22** (x2), 27 (x1) | W25_1 | 61 | JBHNXA000000000 | 78 |
|  |  |  |  | W25_14 | 22 | JBHNWZ000000000 |  |
|  | W26 | 18 | **25** (x5), **36** (x4), 26 (x3), **30** (x2), 28 (x1),  **29** (x1), **61** (x1), 65 (x1) | W26_1 | 29 | JBHNWY000000000 | 72 |
|  |  |  |  | W26_6 | 36 | JBHNWW000000000 |  |
|  |  |  |  | W26_17 | 30 | JBHNWX000000000 |  |
|  | W6 | 19 | **53** (x17), **51** (x1), **57** (x1) | W6_22 | 57 | JBHNWQ000000000 | 100 |
|  |  |  |  | W6_5 | 51 | JBHNWP000000000 |  |

Profiles in bold character were sequenced. F represents the frequency of sequenced isolates in the set of isolates collected from each wine sample.
